#  To what Extent Would PBL be Best Incorporated into a Pharmacy Curriculum? 

**Published:** 2012

**Authors:** Hossein Vatanpour

For the time being our pharmacy curriculum is in Iran mostly based on lecturing methods as instructional strategies. In this method of education a great deal of declarative knowledge as well as a large number of students could be covered at the same time. 

Indeed based on the job description of a pharmacist, pharmacy graduates should work in a relevant field in pharmacy retail, in order to be able to solve any health related problems, rather than having merely declarative knowledge. 

Colleges of pharmacy can utilize problem based learning (PBL) to aid anticipated learning outcomes and practice competencies of entry level pharmacists. Problem-based learning is an effective approach to enhance cognitive strategies and long-term memory as well. In this method pharmacists will be involved in expanded patient care responsibilities which require graduates to possess enhanced communication skills, greater problem-solving capabilities, effective critical thinking abilities and decision making skills.

Moreover, PBL has the potential to integrate the basic sciences with clinical practice too. However, to achieve the PBL method of learning, curriculum modification and various instructional strategies will have to be considered to facilitate the learning outcomes needed for the practice of pharmaceutical care. 

Fortunately, already a number of pharmacy courses includeing, over the counter (OTC) medicines, pharmacotherapeutics, pharmacy administration, toxicology, pharmacy practice, pharmacology, clinical pharmacy and some other advanced courses, lend themselves to the PBL instructions. However, the use of the PBL learning method may in some basic courses such as pharmaceutical sciences, biochemistry, medicinal chemistry and so on face difficulties. For such courses is there another option close to PBL is called the problem-solving model. The differenc between then is that is in PBL the specific situation is defined as the problem and student should analyse the situation and provide a solution, whereas in the problem solving metod for student better understanding problems have been designed for students to solve together and find answers to them. Hence basic sciences in pharmacy education are best taught by conventional curriculum and problem solving method and advanced courses could be taught using the PBL model. In order to succeed, we need to change the pharmacy curriculum, by integrating the knowledge with practice. Conditions needed to make integration possible are as follows: 1) a well- developed national health policy; 2) a plan for the development and coordination of human resources for health; 3) a discussion and decision committee to review the curriculum; and 4) organization to consider financial aspects, compensation, maintenance of quality of the services and academic standards. 

Apart from the face that PBL strategy is valuable in helping students to learn team collaboration, listening, and participating in interdisciplinary discussion, students will be able to clarify for themselves the abilities they bring to a health care team. This will help them to obey their job responsibility as a good consultant for physicians, nurses, patients, and other health care professionals. 

PBL is directing the learning strategy to: keep the students engaged in active learning, hold students responsible for the entire knowledge base that applies to different courses of pharmacy, use a facilitator of knowledge (tutor) rather than presenter of knowledge, and design a variety of clinical scenarios that can be extended into chronic followed-up care and treatment. To achieve this situation it is highly recommended to revise pharmacy curriculum and develop methods of teaching and learning.

*Hossein Vatanpour* *is currently working as a professor at the* *Department of Toxicology and Pharmacology, School of Pharmacy, Shahid Beheshti University of Medical Sciences, Tehran, Iran.* *He could be reached at the following e-mail address:* *vatanpour.hossein@gmail.com.*

